# Comparison of allergens and symptoms in patients with allergic rhinitis between 1990s and 2010s

**DOI:** 10.1186/s13223-020-00455-9

**Published:** 2020-07-01

**Authors:** Ji Heui Kim, Shin Ae Kim, Ja Yoon Ku, Won Ki Cho, Chol Ho Shin

**Affiliations:** 1 Department of Otorhinolaryngology-Head and Neck Surgery, Asan Medical Center, University of Ulsan College of Medicine, 88 Olympic-ro 43-gil, Songpa-gu, Seoul, Republic of Korea; 2 Department of Otorhinolaryngology-Head and Neck Surgery, Soonchunhyang University College of Medicine, Seoul Hospital, Seoul, Republic of Korea

**Keywords:** Allergens, Allergic Rhinitis, Epidemiology

## Abstract

**Background:**

The prevalence of allergic rhinitis (AR), an environment- and lifestyle-dependent condition, has been constantly increasing in Korea. Although the environment and lifestyle of the Korean people have recently undergone rapid changes, corresponding changes in the characteristics of AR patients have not been well documented. Therefore, we aimed to outline the changes in allergens and clinical manifestations of AR in Korean patients from the 1990s and 2010s.

**Methods:**

We reviewed 1447 and 3388 AR patients who visited the same tertiary hospital in the 1990s and 2010s, respectively. All patients were diagnosed with AR based on the presence of characteristic symptoms, positive skin prick test results, and answered a symptom questionnaire at the time of visit. We compared differences in the allergens and results of the symptom questionnaire between the two sets of patients.

**Results:**

When compared with the 1990s, the rate of sensitization to house dust mites, cockroaches, *Aspergillus*, *Alternaria*, and tree pollen significantly increased and that to cat fur significantly decreased in patients from the 2010s (all *P* < 0.05). Male predominance was observed with two peaks in the age distribution of patients from the 2010s. The proportion of patients with moderate-to-severe nasal obstruction and itching of the nose/eye increased (each *P* < 0.05) and that of patients with minor symptoms such as olfactory disturbances, cough, sore throat, and fatigue also increased (all *P* < 0.01) in the 2010s.

**Conclusions:**

Allergen reactivity and type and symptom severity in Korean AR patients significantly varied between the 1990s and 2010s. Our results may therefore be helpful for patient counseling and management.

## Background

Allergic rhinitis (AR) is a common chronic upper airway inflammatory disease. As of 2010, Korea had an AR prevalence of 16.2% [1], which has been continuously increasing [2]. AR is characterized by symptoms such as nasal obstruction, rhinorrhea, sneezing, and itching of the nose/eye, all of which are worse in the morning than during the day [3]. These symptoms can be aggravated by histamine, methacholine, allergens, hypertonic saline, capsaicin, or cold dry air [3, 4]. Although AR patients have been known to complain of symptoms other than those described above, clinicians appear to be unaware of atypical and nonspecific symptoms.

With the rapid development and industrialization of Korea, the environment and lifestyle of its residents have changed considerably in the past 20 years. Since the manifestation of AR is closely dependent on both the environment and lifestyle, its current epidemiology may differ from that of 20 years ago. The house dust mite is the most common allergen for Korean AR patients [1]. However, changes in the distribution of other allergens are not well known. In addition, sex and age distribution may have changed over the span of 20 years. Given that changes in the allergens and clinical manifestations of AR in Korea have not been well reported, we investigated these factors by comparing questionnaire results of patients from the 1990s and 2010s.

## Methods

### Patients

A total of 2722 patients between January 1994 and December 1994 (representing the 1990s) and 4980 patients between January 2010 and December 2014 (representing the 2010s) completed the same questionnaire when they underwent a skin prick test (SPT) at the Asan Medical Center, a tertiary care center in Korea, for an AR diagnosis. Patients with rhinitis were referred to our center when primary or secondary clinics across Korea were unable to adequately treat rhinitis symptoms. The proportion of patients in our hospital living in Seoul and other urban, suburban, or rural regions corresponds to the proportion of Koreans living in Seoul and other regions. All patients had a suggestive clinical history of AR with at least two of the following symptoms: recurrent nasal obstruction, rhinorrhea, sneezing, and itching of the nose/eye with no history of acute upper airway infections. The questionnaire was completed either by the patients themselves (if aged > 14 years) or by their caregivers (if aged ≤ 14 years). This study meets the ethical principles of the Declaration of Helsinki and was approved by the Institutional Review Board of Asan Medical Center (2017-1402), which waived the requirement of patient consent.

The study included 1447 of the 2722 patients from the 1990s and 3388 of the 4980 patients from the 2010s with positive SPT results. Patients were diagnosed with AR if they had at least two of the abovementioned symptoms and positive SPT results. The mean age of the 1990s patients was 24.3 (range 3–72) years and that of the 2010s patients was 33.6 (range 3–84) years.

### Skin prick test

Standardized allergen extracts (Bencard Allergie, München, Germany) including house dust mites (*Dermatophagoides pteronyssinus* [Dp], *D. farinae* [Df]), cat fur, dog dander, *Alternaria*, *Aspergillus*, cockroach, mixed grass pollen, mixed tree pollen, ragweed, and mugwort were used. Histamine and saline were used as positive and negative controls, respectively. If the patients were taking antihistamines, the dose was discontinued for at least a week before conducting SPT. SPT was performed on either or both forearms, depending on patient age. The distance between any two allergens was maintained at 2 cm to avoid cross-contamination. A wheal diameter measuring > 3 mm indicated a positive response to the allergen.

### Questionnaire

The questionnaire designed by the authors consisted of the following items: symptoms, the season with the most severe symptoms, the time of the day with the most severe symptoms, and aggravating factors for the symptoms.

### Statistical analysis

The characteristics of AR patients from the 1990s and 2010s were compared using the Chi square test. Data were analyzed using Statistical Package for Social Sciences (SPSS) 21 (IBM, Chicago, IL, USA). *P* < 0.05 was considered statistically significant.

## Results

### Allergens

Dp and Df were the most common allergens in both patient groups. However, the proportions of positive SPT results for Dp and Df were significantly higher in patients from the 2010s than in those from the 1990s (*P* = 0.016 and *P* = 0.022, respectively). In addition, the proportions of positive rates for tree pollen as well as those for indoor allergens such as cockroach, *Aspergillus*, and *Alternaria* were significantly higher in patients from the 2010s (*P* < 0.05 for each). In contrast, the proportions of positive results for cat fur was lower in patients from the 2010s than in those in the 1990s (*P* < 0.001) (Table 1).

**Table 1:** Positive reactivity to allergens in the skin prick test

Allergens	Number of patients (%)		P*
	1990s (n = 1447)	2010s (n = 3388)	
*Dermatophagoides pteronyssinus*	1001 (62.9)	2458 (72.6)	0.016
*Dermatophagoides farinae*	964 (66.6)	2378 (70.2)	0.022
Cat fur	490 (33.9)	856 (25.3)	< 0.001
Dog dander	343 (23.7)	772 (22.8)	0.502
Cockroach	192 (13.3)	775 (22.9)	< 0.001
*Aspergillus*	37 (2.6)	271 (8.0)	< 0.001
*Alternaria*	70 (4.8)	224 (6.6)	0.019
Tree pollen	202 (14.0)	697 (20.6)	< 0.001
Mugwort	280 (19.4)	670 (19.8)	0.733
Ragweed	175 (12.1)	473 (14.0)	0.081
Grass pollen	124 (8.6)	243 (7.2)	0.097

* Chi square test

The proportion of polysensitized patients was significantly higher in the 2010s (59%) than in the 1990s (55.6%) (*P* = 0.030). Most patients who were sensitized to pollen in both the 1990s and 2010s showed a positive response to other allergens such as Dp, Df, cat fur, dog hair, cockroach, *Aspergillus*, and *Alternaria*. However, the proportion of patients sensitized only to pollens was significantly lower in the 2010s (6.8%) than in the 1990s (10.5%) (*P* < 0.001).

### Sex and age

**Fig. 1:**
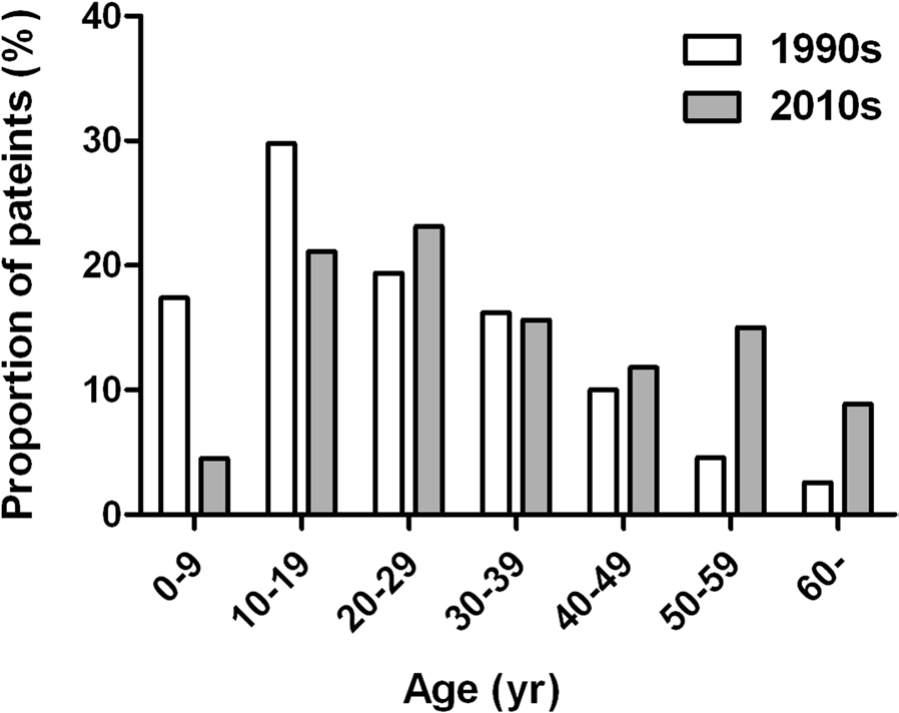
Age distribution of patients with allergic rhinitis from the 1990s and 2010s

The male-to-female ratio of AR patients significantly increased, from 1.41 (847:600) in the 1990s to 1.78 (2169:1219) in the 2010s (*P *< 0.001). In the 1990s, AR prevalence was the highest in patients aged 10–19 years (29.8%) and gradually decreased with age. In contrast, in the 2010s, the prevalence was the highest in patients aged 20–29 years (23.1%) and 10–19 years (21.1%), gradually decreasing and then increasing again in those aged 50–59 years (15%) (Fig. 1).

### Symptoms

The most common symptom was nasal obstruction, followed by rhinorrhea, sneezing, and itching in patients from both the 1990s and 2010s (Table 2). In the 2010s, the proportion of rhinorrhea was slightly lower than in the 1990s (*P* = 0.030), whereas the proportion of itching was higher (*P* < 0.001). When the severity of major symptoms was compared between the 1990s and 2010s, the proportions of moderate-to-severe nasal obstruction and itching were higher in the 2010s, whereas that of rhinorrhea was lower and that of sneezing remained unchanged (Table 3). Among minor symptoms, the rates of olfactory disturbance, cough, sore throat, and fatigue were significantly higher in the 2010s than in the 1990s (all *P* < 0.01, Table 2). In patients allergic to Dp, Df, cat fur, dog dander, and tree pollen, the most common symptoms were nasal obstruction, followed by rhinorrhea, sneezing, and itching. The proportions of nasal obstruction, rhinorrhea, and sneezing did not differ between the 1990s and 2010s, whereas the proportion of itching was higher in the 2010s than in the 1990s (Table 4). However, the proportions of symptoms severity based on allergen differed between the 1990s and 2010s. The proportions of moderate-to-severe nasal obstruction and itching in patients allergic to Dp, Df, cat fur, and dog dander were higher in the 2010s than in the 1990s. The proportion of moderate-to-severe rhinorrhea in patients allergic to Dp and Df was lower in the 2010s than in the 1990s and that of moderate-to-severe sneezing in patients allergic to Dp, Df, cat fur, dog dander, and tree pollen did not differ between the 1990s and 2010s (Table 4).

**Table 2:** Comparison of patient symptoms between the 1990s and 2010s

Symptoms	Number of patients (%)		P*
	1990s (n = 1447)	2010s (n = 3388)	
Sneezing	1121 (77.5)	2568 (75.8)	0.210
Rhinorrhea	1239 (85.6)	2815 (83.1)	0.028
Nasal obstruction	1299 (89.8)	3078 (90.9)	0.241
Itching of the nose/eye	702 (48.5)	2027 (59.8)	< 0.001
Headache	798 (55.1)	1846 (54.5)	0.672
Post nasal drip	1025 (70.8)	2330 (68.8)	0.154
Olfactory disturbance	192 (13.3)	587 (17.3)	< 0.001
Cough	319 (22.0)	945 (27.9)	< 0.001
Sore throat	227 (15.7)	659 (19.5)	0.007
Fatigue	201 (13.9)	577 (17.0)	< 0.001

* Chi square test

**Table 3:** Severity of symptoms in patients from the 1990s and 2010s

Symptoms	Number of patients (%)		P*
	1990s (n = 1447)	2010s (n = 3388)	
Nasal obstruction			0.003
Mild	555 (38.4)	1148 (33.9)	
Moderate to severe	892 (61.6)	2240 (66.1)	
Rhinorrhea			< 0.001
Mild	926 (64.0)	2351 (69.4)	
Moderate to severe	521 (36.0)	1037 (30.6)	
Sneezing			0.162
Mild	933 (64.5)	2255 (66.6)	
Moderate to severe	514 (35.5)	1133 (33.4)	
Itching of the nose/eye			< 0.001
Mild	981 (67.8)	2010 (59.3)	
Moderate to severe	466 (32.2)	1378 (40.7)	

* Chi square test

**Table 4:** Comparison between allergens, symptoms, and severity of symptoms in patients from the 1990s and 2010s

Allergen	Proportion of symptoms (%)											
	Nasal obstruction			Rhinorrhea			Sneezing			Itching of the nose/eye		
	1990s	2010s	*P*	1990s	2010s	*P*	1990s	2010s	*P*	1990s	2010s	*P*
*Dermatophagoides pteronyssinus*	91.2	92.7	0.132	85.8	83.9	0.159	78.8	77.1	0.293	50.3	61.4	< 0.001
*Dermatophagoides farinae*	90.8	92.1	0.227	85.5	83.5	0.143	79.0	77.1	0.222	49.0	60.8	< 0.001
Cat fur	92.7	93.0	0.817	89.4	84.7	0.016	81.4	77.3	0.077	52.9	63.9	< 0.001
Dog dander	93.6	93.5	0.969	87.5	87.3	0.942	81.6	80.2	0.571	52.8	65.0	< 0.001
Tree pollen	86.1	92.4	0.006	85.6	85.2	0.882	84.7	77.6	0.030	49.0	65.7	< 0.001

Allergen	Proportion of moderate-to-severe symptoms (%)											
	Nasal obstruction			Rhinorrhea			Sneezing			Itching of the nose/eye		
	1990s	2010s	*P*	1990s	2010s	*P*	1990s	2010s	*P*	1990s	2010s	*P*
*Dermatophagoides pteronyssinus*	63.7	70.2	<0.001	35.0	30.2	0.006	34.7	35.7	0.605	32.8	43.0	< 0.001
*Dermatophagoides farinae*	63.7	69.2	0.002	34.4	30.3	0.018	35.1	34.8	0.847	32.2	42.1	< 0.001
Cat fur	67.3	69.9	0.338	38.2	33.6	0.095	39.0	38.8	0.944	35.3	45.8	< 0.001
Dog dander	72.0	71.8	0.932	36.4	36.5	0.978	36.2	39.0	0.368	37.0	47.8	0.002
Tree pollen	57.9	70.3	0.001	33.7	36.6	0.446	41.1	37.7	0.388	32.2	44.0	0.003

* Chi square test

Patients experienced the most severe symptoms during seasonal changes, followed by throughout the year and winter in both the 1990s and 2010s (Table 5). When compared with the 1990s, reported symptom severity increased during seasonal changes (*P* < 0.001), decreased throughout the year (*P* = 0.003), and remained unchanged during individual seasons (*P* > 0.05) in the 2010s. The time of the day with the most severe symptoms was the morning, followed by throughout the day in both the 1990s and 2010s. In the 2010s, the proportion of patients who reported the most severe symptoms throughout the day was significantly higher than that in the 1990s (*P* < 0.001). However, reports of severity in the morning were lower in patients from the 2010s than in those from the 1990s (*P* < 0.001).

**Table 5:** Comparison of seasons and times of day with most severe symptoms between 1990s and 2010s

	Number of patients (%)		P*
	1990s (n = 1447)	2010s (n = 3388)	
Season			
Spring	101 (7.0)	226 (6.7)	0.695
Summer	38 (2.6)	119 (3.5)	0.111
Fall	52 (3.6)	110 (3.2)	0.539
Winter	288 (19.9)	659 (19.5)	0.717
The change of seasons	456 (31.5)	1275 (37.6)	< 0.001
Throughout the year	454 (31.4)	918 (27.1)	0.003
No answer	48 (3.3)	81 (2.4)	N/A
Time			
Morning	637 (44.0)	1158 (34.2)	< 0.001
Daytime	101 (7.0)	255 (7.5)	0.505
Evening	211 (14.6)	572 (16.9)	0.050
Bedtime	168 (11.6)	412 (12.2)	0.590
Throughout the day	292 (20.2)	900 (26.6)	< 0.001
No answer	38 (2.6)	91 (2.7)	N/A

* Chi square test; N/A, not available

Dust and cold air commonly aggravated AR symptoms in patients both from the 1990s and 2010s (Table 6). Symptom aggravation due to cleaning, dust, bedding, and air conditioning was more common in the 2010s than in the 1990s (all *P* < 0.001).

**Table 6:** Comparison of aggravating factors of AR symptoms between patients from the 1990s and 2010s

	Number of patients (%)		P*
	1990s (n = 1447)	2010s (n = 3388)	
Cleaning	111 (7.7)	542 (16.0)	< 0.001
Dust	538 (37.2)	1913 (56.5)	< 0.001
Bedding	58 (4.0)	355 (10.5)	< 0.001
Smoking	192 (13.3)	430 (12.7)	0.606
Cold air	632 (43.7)	1400 (41.3)	0.135
Air conditioner	258 (17.8)	961 (28.4)	< 0.001

* Chi square test

## Discussion

In this study, we report that patient reactivity to indoor allergens and tree pollen as well as the proportion of male AR patients have increased over the past 20 years in Korea, with peaks in the 10–29 years and 50–59 years age groups. Furthermore, the proportions of patients with moderate-to-severe nasal obstruction, itching of the eye/nose, and minor symptoms have increased. These findings indicate that allergen reactivity and characteristics of AR patients have changed with the industrialization and urbanization of Korea.

AR is an airway disease showing increasing prevalence owing to industrialization and an increase in air pollutants, both of which are important environmental and external etiologic factors [5, 6]. In Korea, the prevalence of AR has been increasing with corresponding changes in air pollution, pollen distribution, westernized lifestyles, and regional environmental conditions [7, 8]. This study showed that indoor allergens are the most common, with sensitization rates having increased over the last 20 years. During this time, the rates of sensitization to tree pollen and polysensitization have also increased. These results mirror those reported in Western countries, which have also experienced similarly increasing rates of industrialization and urbanization. According to a United States of America health survey, allergic skin test reactivity is more common in urban areas than in rural areas [9]. Although indoor allergens such as house dust mites, molds, insects (cockroaches), and animal dander are most common in AR patients [3], an increase in sensitization to tree pollen is also noteworthy. One study reported that the rate of sensitization to pollen in Korean children with AR was 37% and increased with age, contributing to the overall increase in AR prevalence in Korea [10]. Another recent Korean study reported an increase in pollen concentration from 2012 to 2016, implicating it as a cause of higher numbers of AR patients in the fall [11].

Male sex has been reported as a risk factor for AR in Korea [1]. In line with this report, our study showed that the proportion of male AR patients was higher in the 2010s than in the 1990s, suggesting male sex susceptibility to AR. In the 2010s, the age distribution of AR patients showed two peaks, one at 10–29 years and another at 50–59 years. This finding was different from that in Chinese patients, which showed only one peak at 10–14 years and decreased with age [12]. This difference may be due to different regional environmental conditions.

In both the 1990s and 2010s, the most common symptom among the four major AR symptoms was found to be nasal obstruction, with > 60% of patients experiencing moderate-to-severe nasal obstruction. This implies that many AR patients visit the hospital because of a late response, which in the case of AR is characterized by prolonged nasal obstruction. When the relationship between allergen and symptoms was analyzed, more than 85% of the patients allergic to common allergens such as Dp, Df, cat fur, dog dander, and tree pollen complained of nasal obstruction and rhinorrhea; the proportions of nasal obstruction, rhinorrhea, and sneezing did not differ between the 1990s and 2010s. However, the proportions of moderate-to-severe nasal obstruction and itching were higher in the 2010s than in the 1990s, which was owing to the increase in patients allergic to the most common allergens such as Dp and Df. The decrease in moderate-to-severe rhinorrhea and lack of change in sneezing were also determined by the relationship between symptoms and Dp and Df.

More than a third of the patients experienced symptoms during seasonal changes, and this proportion was higher in the 2010s than in the 1990s. The most common season during which symptoms occurred was winter, accounting for approximately 20% of patients in both the 1990s and 2010s. This observation of cold air worsening symptoms in approximately 40% of patients indicates that cold air induces nasal obstruction and rhinorrhea. Cold air can damage mast or epithelial cells, stimulate and depolarize trigeminal sensory nerves, and induce axon responses and parasympathetic reflexes [3, 13, 14]. As the lifestyle of Koreans becomes more westernized, the use of bedding and air conditioners increases, resulting in more common and severe AR symptoms in the 2010s than in the 1990s.

The results of this study did not accurately represent the characteristics of AR patients in the general Korean population as it was conducted at a tertiary care center. Moreover, the study did not reflect existing regional differences within Korea. However, as most of the patients who visited our center were not only from major cities but also from other rural or suburban regions, the results should roughly reflect the characteristics of AR patients across Korea. Another limitation of this study is that it had a relatively small sample size, with 3388 patients sampled across 3 years in the 2010s and 1447 patients sampled across a year in the 1990s. In actuality, the number of AR patients did not decrease but rather increased. This discordance between sample size and the actual number of AR patients is caused by a recent increase in the measurement of specific immunoglobulin E to identify sensitized allergens. Because the sensitivity and specificity for SPT was 85% and 77%, respectively, according to a recent systematic review and meta-analysis [15], our study may have missed existing AR patients or incorrectly included non-AR patients. Nevertheless, this limitation was overcome by the overall large sample size.

## Conclusions

When compared with the 1990s, positive reactivity to indoor allergens and tree pollen was higher in the 2010s, and the proportion of male AR patients also increased, with two peaks in the age distribution. In addition, the proportion of AR patients with moderate-to-severe nasal obstruction, itching of the eye/nose, and minor symptoms such as olfactory disturbance, cough, sore throat, and fatigue increased in the 2010s. Our results may reflect changes in environmental conditions and lifestyle in Korea and can be helpful for patient counseling and management.

## Data Availability

Not applicable.

## Abbreviations

AR: Allergic rhinitis
SPT: Skin prick test
*Dermatophagoides pteronyssinus*: Dp
*D. farina*: Df
